# Ballistic research techniques: visualizing gunshot wounding patterns

**DOI:** 10.1007/s00414-020-02265-5

**Published:** 2020-02-14

**Authors:** Tom Stevenson, Debra J. Carr, Karl Harrison, Richard Critchley, Iain E. Gibb, Sarah A. Stapley

**Affiliations:** 1 Impact and Armour Group, Centre for Defence Engineering, Cranfield University, Defence Academy of the United Kingdom, Shrivenham, SN6 8LA UK; 2 Cranfield Forensic Institute, Cranfield University, Defence Academy of the United Kingdom, Shrivenham, SN6 8LA UK; 3 Present Address: Defence and Security Accelerator, Porton Down, Salisbury, SP4 0JQ UK; 4 Centre for Defence Radiology, at c/o Sickbay, HMS Nelson, HMNB Portsmouth, Hampshire, PO1 3HH UK; 5 Royal Centre for Defence Medicine, ICT Building, Research Park, St Vincent Drive, Birmingham, B15 2SQ UK

**Keywords:** Gunshot, Wound, Limb, X-ray, Ultrasound, CT

## Abstract

There are difficulties associated with mapping gunshot wound (GSW) patterns within opaque models. Depending on the damage measurement parameters required, there are multiple techniques that can provide methods of “seeing” the GSW pattern within an opaque model. The aim of this paper was to test several of these techniques within a cadaveric animal limb model to determine the most effective. The techniques of interest were flash X-ray, ultrasound, physical dissection, and computed-tomography (CT). Fallow deer hind limbs were chosen for the model with four limbs used for each technique tested. Quarantined 7.62 × 39 mm ammunition was used for each shot, and each limb was only shot once, on an outdoor range with shots impacting at muzzle velocity. Flash X-ray provided evidence of yaw within the limb during the projectile’s flight; ultrasound though able to visualise the GSW track, was too subjective and was abandoned; dissection proved too unreliable due to the tissue being cadaveric so also too subjective; and lastly, CT with contrast provided excellent imaging in multiple viewing planes and 3D image reconstruction; this allowed versatile measurement of the GSW pattern to collect dimensions of damage as required. Of the different techniques examined in this study, CT with contrast proved the most effective to allow precise GSW pattern analysis within a cadaveric animal limb model. These findings may be beneficial to others wishing to undertake further ballistic study both within clinical and forensic fields.

## Introduction

Damage caused to a target by the impact of a projectile in research can be measured in a number of ways, for example, depth of penetration (DoP), kinetic energy (KE) transfer, or calculation of area or volume of damage [1–12]. One of the challenges associated with gathering such data is to optimise the method(s) used for the target material under study. The last century has seen the use of target materials for ballistic research including, but not limited to, soap, gelatine, cadaveric human tissue, cadaveric animal tissue, and live animal tissue [13].

With synthetic models such as gelatine, the relative transparency allows for visual analysis of gunshot wounding (GSW) using techniques such as high speed video (HSV) to capture the effect of the projectile on the target in real time [6, 10, 12, 14]. With respect to the study of GSW in cadaveric or live tissue, one of the difficulties in the analysis of wounding patterns is the opacity of the surrogate.

This paper examines several techniques to ascertain the most effective method to measure GSW patterns in a cadaveric animal model.

### Flash X-ray

Flash X-ray is a relatively expensive, non-portable method of capturing an image via a small dose of radiation. The use of flash X-ray allows a snapshot of what happens within opaque tissue during the ballistic event under study. With knowledge of the timing of imaging in relation to the projectile’s position within or outside of the model, measurements of temporary cavity dimensions can be captured, as well as evidence of bone fracture, and yaw of the projectile [15–20].

### Ultrasound

Ultrasound is a relatively cheap, portable, quick, and non-invasive method of imaging within human or animal tissues (or synthetic materials). It also offers a non-irradiating method of imaging to try and visualise a GSW track within the target. Operation of ultrasound requires specialist training with challenges of interpreting images including orientation of static images without a reference point. Ultrasound is disrupted and images distorted by gas. This is minimised at the skin surface with a gel interface but gas within any wound tracts means that deeper imaging is impossible and accurate dimensions cannot be measured. Within the clinical setting, ultrasound has been used with regard to GSW to determine the extent of internal haemorrhage or free fluid associated with thoracic, abdominal, and pelvic injury to assist the decision-making process towards rapid surgical intervention [21]. With regard to mapping GSW tracks, the literature appears limited with examples of a case report [22] and a live animal model study [23]. There has been an increasing use of ballistic gelatine in models for ultrasound training, such as vessel cannulation or joint injection [24–28].

### Dissection

Physical dissection remains a method to lay open a GSW track and allow direct visualisation of the tissues. The main disadvantage is that the tissue under study will be destroyed by dissection. GSWs are usually managed surgically within the clinical arena. Debate about the extent of surgical tissue debridement persists, with contrasting arguments for either greater or less tissue excision proposed (e.g. [29–36]). With regard to investigating GSW in experiments, expert clinicians would frequently be used to excise damaged tissue. The total mass of excised tissue is then used as a measure of wounding severity [37–40]. Another use of excised tissue has been to determine the morphology of cells within the zone of injury, identify the border of damaged versus undamaged cells, or to determine the reversible or non-reversible changes seen with serial measurements over nominated time intervals [16–18, 38, 41–43]. With regard to this study, tissue viability was not under investigation as the animal tissue in question was cadaveric.

### Computed-tomography

As a radiological modality, computed-tomography (CT) is neither cheap, nor easily portable, and requires expert interpretation of images produced. CT provides an in-depth and detailed method to precisely study the anatomy of tissues which would otherwise be obscured from view. CT is employed to delineate the path taken by projectiles, such as bullets, through tissues in the acute clinical setting and in forensic examinations [44–47]. For the purposes of this study, a method was developed to inject contrast into the wound tracks which allowed for multi-planar reconstruction (MPR) and 3D reconstructed images for further analysis and can be found in more detail at [48]. This method was also utilised in recent work examining the effect of military clothing on GSW patterns in a cadaveric deer limb model [49].

## Materials and methods

Ethical approval for this work was granted through the Cranfield University Research Ethics System (CURES/3579/2017).

### Materials

**Fig. 1:**
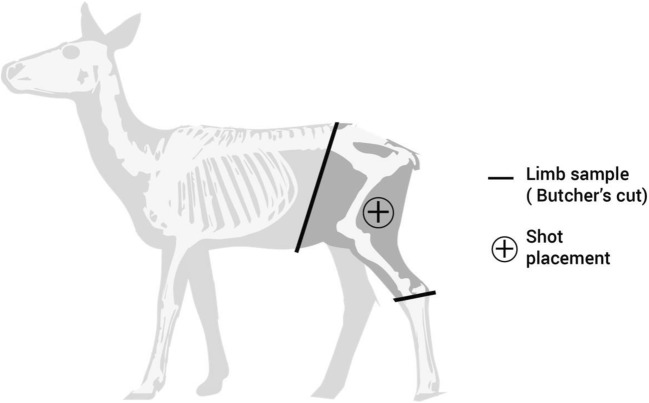
Fallow deer anatomy schematic demonstrating limb preparation and shot placement

Fallow deer (*Dama dama*) hind limbs were used in this work.^1^ The similarity in morphology between deer femur bones and human femurs has been discussed [50], and it can be assumed that the soft tissue morphology is equally comparable. The use of fallow deer limbs as a human tissue surrogate was also investigated and discussed in previous work [49]. Limb masses were 11–13 kg and measured approximately 280 mm × 700 mm × 100 mm (width × height × thickness), and were sectioned from the main carcass at the pelvis and the ankle (Fig. 1). The limbs were used as either fresh targets (within 72 hours of culling) or after being stored by freezing and defrosted before use, depending on access to the ballistic test facilities and CT scanner, and availability of the target material. This latter method was required if limbs were obtained outside of the time frame where the test facilities were then subsequently available, where limbs could only be obtained during certain months of the year when the animals were culled. Previous work has suggested that the difference in ballistic wounding to fresh versus defrosted tissues is likely to be negligible [51]. Limbs were examined either during or after shooting using flash X-ray, ultrasound, dissection, or CT (*n* = 4 limbs for each technique). All limbs were shaved over the lateral surface prior to testing.

The ammunition used was from a single batch of 7.62 × 39 mm (7.62 × 39 mm Wolf Hunting Cartridges; lead core, 122 grain full metal jacket, Lot number F-570, made in Russia, 2006). This ammunition type was a typical example faced by UK military service personnel throughout the most recent conflicts in Iraq and Afghanistan [10, 12, 52, 53].

### Methods

**Fig. 2:**
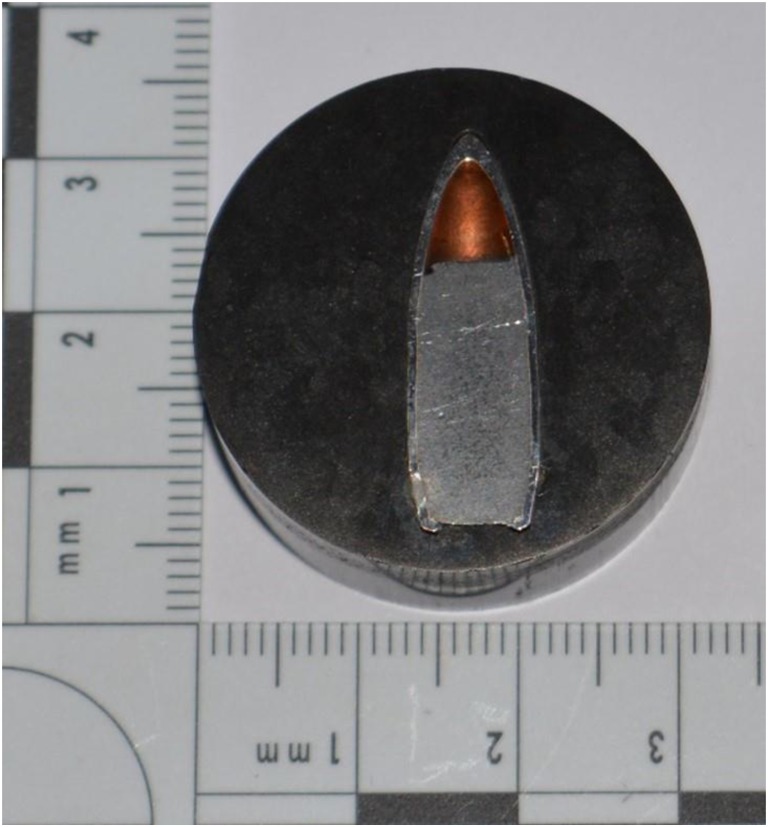
Mounted section of 7.62-mm projectile. Mean core hardness 7.8Hv (SD 0.6Hv, *n* = 3), lead mixed with antimony. Mean jacket hardness of 184.4Hv (SD 12.3Hv, *n* = 3), steel with internal and external copper washes [12]

Ammunition physical and mechanical properties were determined in a previous study (Fig. 2, [12]).

**Fig. 3:**
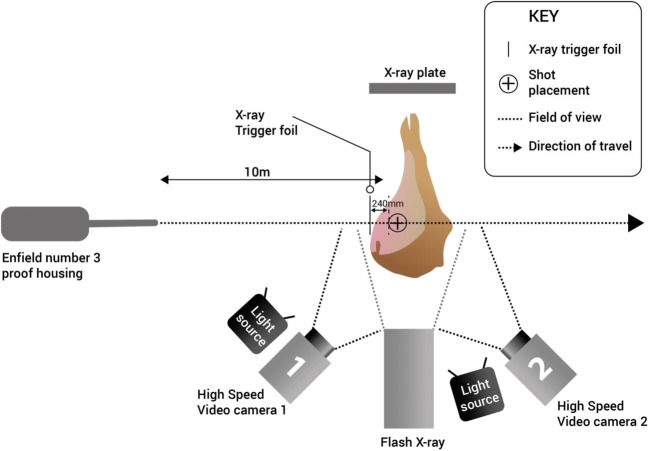
Experimental range set up including flash X-ray positioning (HSV camera 1: Phantom V12 video camera, frames per second = 28,000, shutter speed = 4 μs, resolution = 512 × 384; HSV camera 2: Phantom V1212 video camera, frames per second = 37,000, shutter speed = 5 μs, resolution = 512 × 384)

Shots were taken using Enfield number 3 proof housing fitted with an appropriate barrel from a range of 10 m with two high speed video (HSV) cameras used to capture the event of the entrance and exit of the projectile through the limb (Fig. 3).^2^ Each limb was shot once through the shaved lateral surface of the limb to traverse the posterior thigh soft tissue muscle group.

### Flash X-ray

Flash X-ray (Scandiflash XT 150, Serial No. 320184) was utilised in an attempt to capture the projectile mid-way through the deer limb to determine if the projectile yawed away in the vertical plane from its central axis or not. Flash X-ray strength was 150 kV for all shots, with the X-ray heads situated 2 m from the target, and the exposure plates as close to the target as able. The trigger foil was placed 240 mm in front of the target’s centre, and X-ray exposure time was 35 ns for each use (Fig. 3).

### Ultrasound

**Fig. 4:**
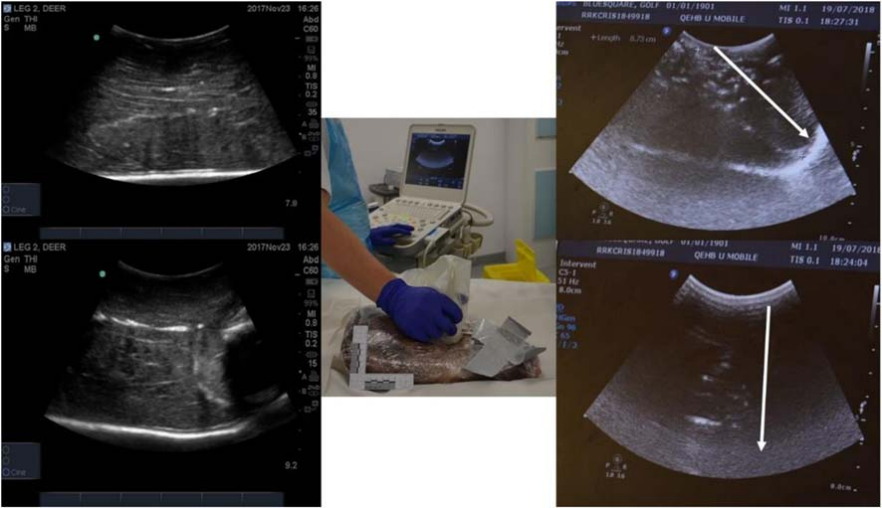
Left, top, and bottom—pre-contrast, pre-shoot ultrasound images; centre—ultrasound in progress, demonstrating curvilinear probe compression of limb soft tissue; right, top, and bottom—post contrast injection ultrasound, highlighted areas represent GSW track, arrows indicate projectile direction of travel

Limbs underwent ultrasonography before and after shooting using a Sonosite M-Turbo ultrasound machine (FUJIFILM Sonosite Ltd., Bedford, UK) with a L38X 10–5 MHz transducer and a C60 curvilinear 5–2 MHz transducer. Images of the wound tracks, when delineated, were measured with inbuilt digital callipers. Ultrasound was also used to scan the limbs immediately prior to CT scanning technique, both before and after contrast injection (Fig. 4). Scanning after the installation of contrast was performed to mitigate for the presence of gas within the tract.

### Dissection

Following shooting, limbs were dissected to measure features of the GSW track, such as track length and width using a steel ruler, and to provide general comment on any other physical properties of the wounds seen, such as evidence of projectile fragmentation.

### Computed-tomography

CT was undertaken for limbs post shooting. With the availability of the scanner being limited to out-of-hours periods due to clinical use, limbs were frozen immediately after shooting until 72 hours prior to the scan date when they were then defrosted. The scanner used was a dual source (2 × 64 slice) Siemens SOMATOM Definition MSCT scanner (System SOMATOM Definition AS, 64622, Siemens AG, Wittelsbacherplatz, DE – 80333 Munchen, Germany). Scans using a standard adult pelvis protocol (exposure figures were 120 kV and 25–32 mAs) with 1.0-mm slice soft tissue and bony reconstructions in the axial, sagittal, and coronal planes. The limbs were wrapped in Clingfilm and scanned initially in situ without contrast. For each limb, a small hole was then made in the Clingfilm over the entrance wound and 10–20 mls Omnipaque 300 contrast (OMNI300, GE Healthcare) was subsequently injected whilst simultaneously probing the wound track via a 5-in. mixing tube connected to a 50-ml Omnifix Luer Lock Solo syringe. The volume range was because contrast was injected via the entrance wound until it could be seen starting to ooze out of the exit wound, then injection stopped with no further contrast added. The entry hole in the Clingfilm was then sealed with duct tape to prevent leakage of the contrast, and the limb re-scanned. The images were reviewed and reconstructed as multiplanar (MPR) and 3D reformats within AGFA Enterprise Imaging Patient Archive and Communications System (PACS) and as part of the Syngo CT2012B software package provided with the CT workstation [48].

### Analysis

**Fig. 5:**
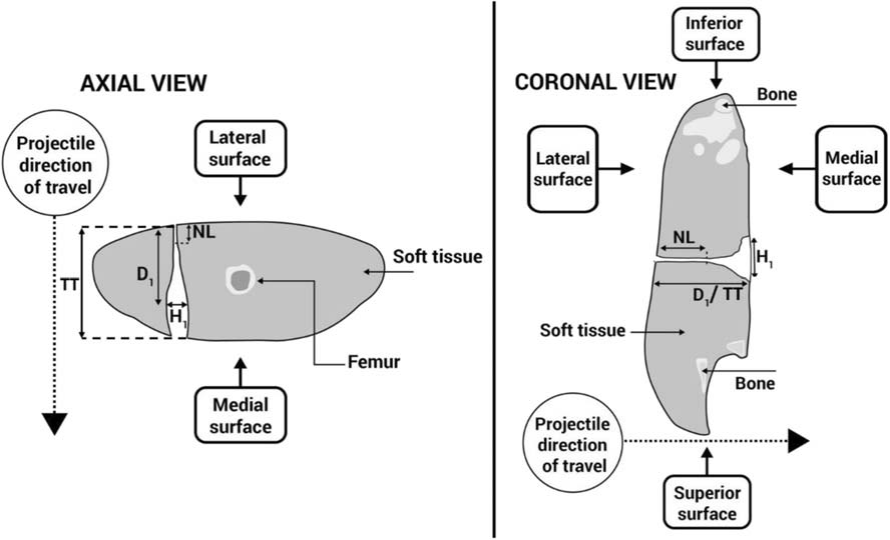
Schematic demonstrating CT scan measurements taken in axial and coronal planes of view (in this example schematic, D1 and TT in the coronal view were the same; however, this varied amongst specimens)

Analysis for each technique was qualitative (and quantitative where possible) with advantages and disadvantages towards use of each considered. Attempted measurements from the wound patterns seen included a neck length or initial narrow section of the wound channel seen (NL), the maximum height of the permanent cavity (H1), the distance from entry to that maximum height (D1), and lastly, the total track length (TT) as well as any other relevant features for comment. Examples of the quantitative measurements taken are shown in Fig. 5.

## Results

Projectiles for all shots had a mean velocity of 735 m/s (SD = 6.6 m/s). All shots perforated with no retained projectiles or projectile fragments within limbs, and with no bone impacts.

### Flash X-ray

**Fig. 6:**
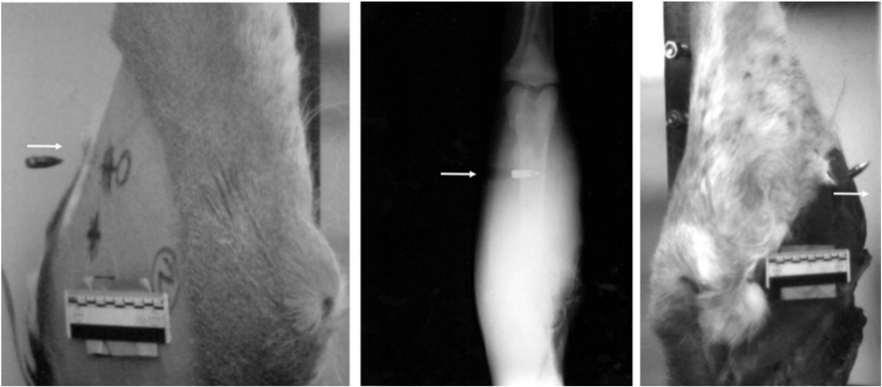
Arrow indicates projectile direction of travel—left: oblique view of front face of deer limb with 7.62 mm projectile about to strike symmetrically; middle: flash X-ray imaging demonstrating 7.62 mm projectile travelling through suspended deer limb, yawing slightly; right: oblique view of rear face of deer limb with 7.62 mm projectile exiting deer limb, yawing significantly

**Fig. 7:**
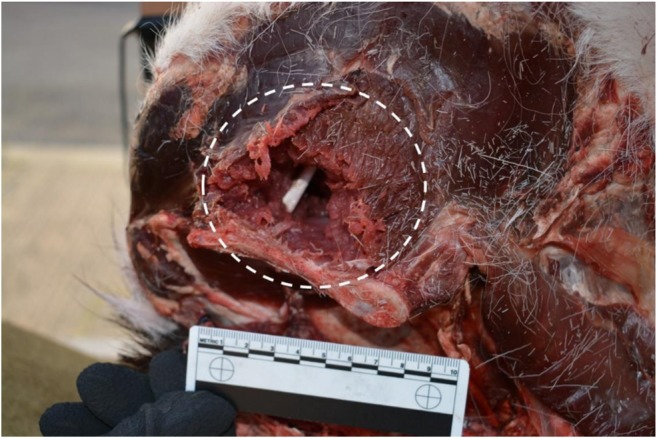
Example of large exit wound seen following yawing projectile exit the deer limb, indicated by dotted circle

Flash X-ray successfully captured the projectile travelling mid-way through the target with all four limbs. Qualitative examination of the HSV footage determined if projectiles would strike the target symmetrically and exit with any significant yaw (Fig. 6); the flash x-ray was able to complement this by demonstrating the yaw as the projectile passed through the mid-point of the limb (Fig. 6). Entrance wounds were small and symmetrical; however, exit wounds were much larger and more varied (Fig. 7). No further measurements could be taken with regard to the wounding pattern dimensions using flash x-ray.

### Ultrasound

No reliable, repeatable measurements of wound track dimensions could be taken from the deer limbs using ultrasound with both intra- and inter-observer variability present. Image quality was variable. The cadaveric musculature appeared homogenously echogenic, making it difficult to identify or measure obvious tissue damage. Wound tracks were difficult to identify unless they had significant gas present, or had contrast material injected to help delineate the GSW track from the other tissues (Fig. 4), and then precise measurement was not possible as the gas prevented deeper visualisation and therefore measurement.

### Dissection

**Fig. 8:**
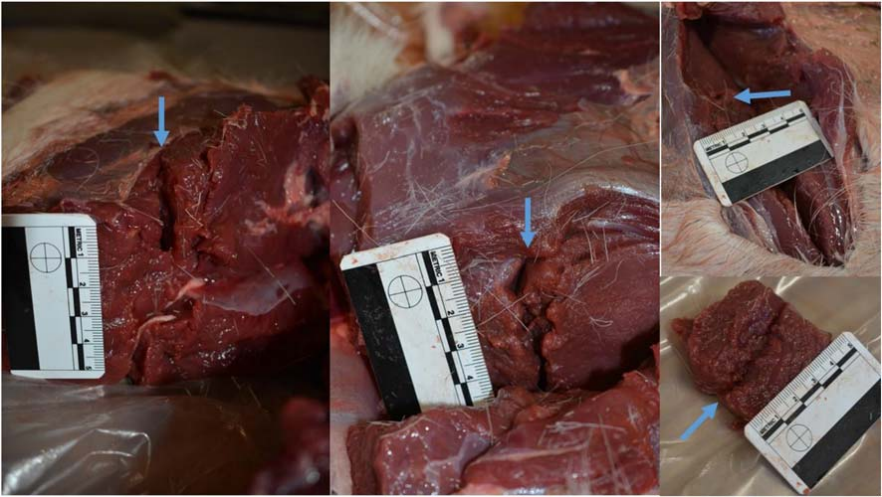
Dissected tissues of cadaveric deer limb, blue arrows point at the GSW track in situ

Of the four limbs which underwent dissection, total track (TT) lengths were measured and recorded in Table 1, and GSW tracks were laid open. Dissection was carried out within 2 h of shooting. All projectiles had perforated the deer limbs through a single wound track, with no physical evidence of secondary fragmentation tracks and no projectile fragmentation recovered. Although this study was of the soft tissue, it was noted that there were no bone fractures, either direct or indirect, that were sustained in any limb. Due to the cadaveric nature of the model, tissue viability could not be examined (Fig. 8). No other reliable or repeatable measurements of wound pattern dimensions could be taken. All limbs were destroyed following dissection.

**Table 1:** Deer limb total track length measurements with mean, SD, and CV

Deer limb number	TT (mm)
1	108
2	96
3	90
4	102
Mean	99
SD	7.7
CV	7.8

### Computed-tomography

**Fig. 9:**
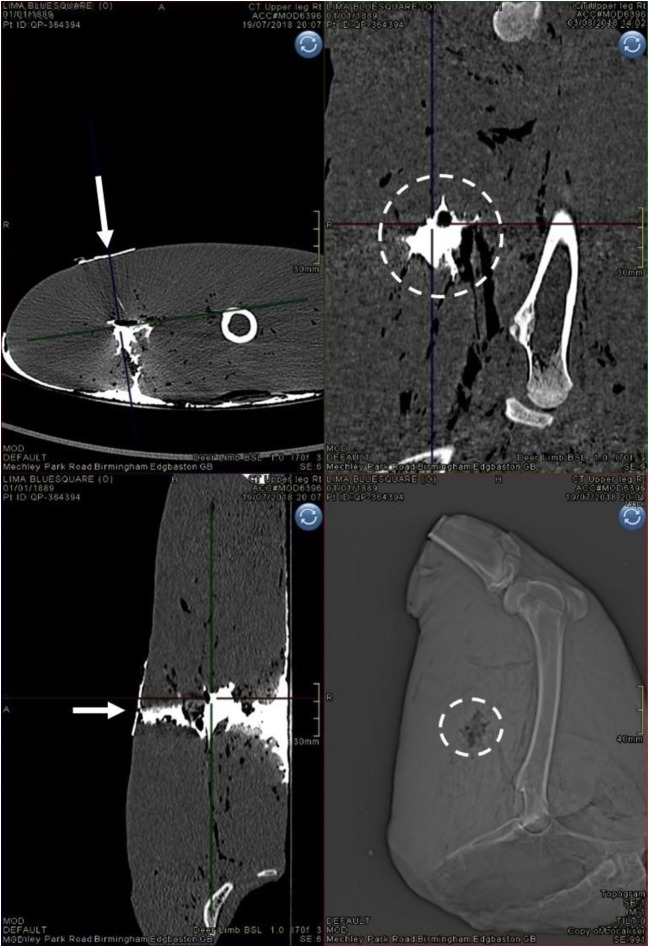
Arrows indicate projectile direction of travel, dotted circles indicate coronal section view of GSW track—clockwise from top left—contrast image, axial plane; contrast image, sagittal plane; CT scout view prior to contrast injection, sagittal plane; contrast image, coronal plane

**Fig. 10:**
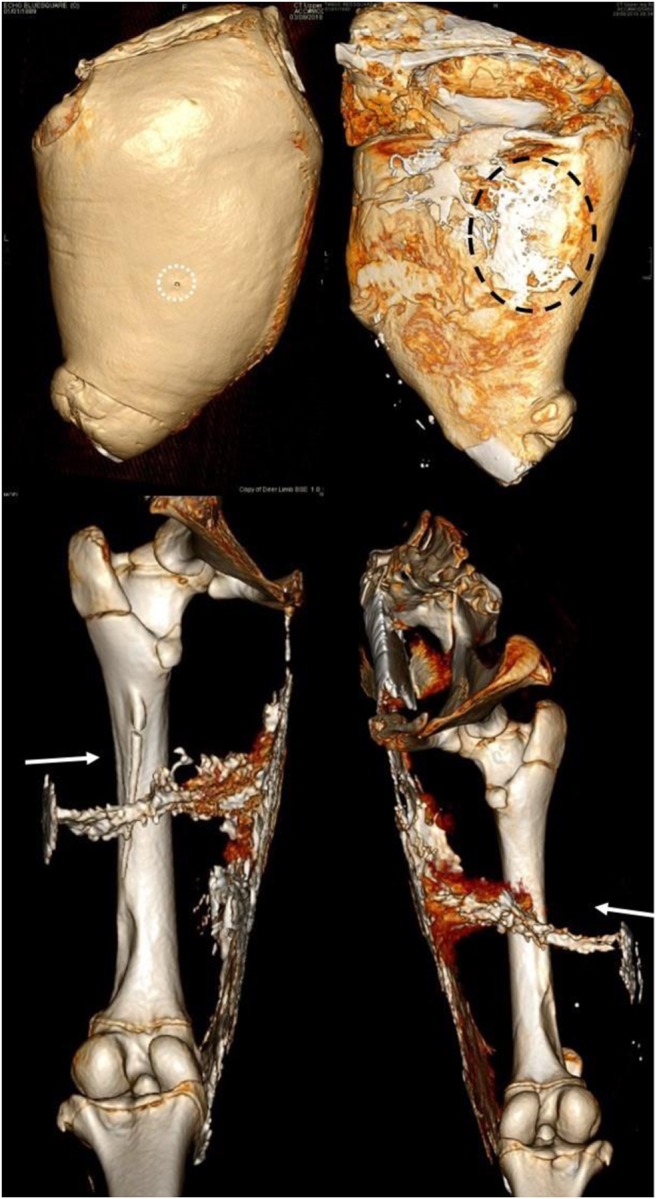
3D reconstructed images, arrows indicate projectile direction of travel, white dotted circle indicates entrance wound, black dotted circle indicates exit wound—clockwise from top left: front face of deer limb without digital subtraction, rear face without digital subtraction, right limb wound profile, left limb wound profile

**Fig. 11:**
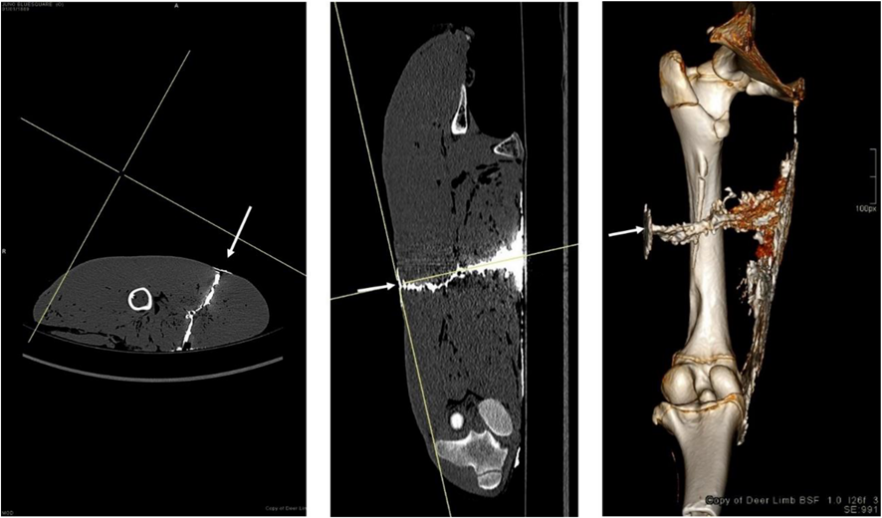
Arrows indicate projectile direction of travel—left: axial view with contrast; middle: coronal view with contrast; right: corresponding 3D reconstruction image in coronal view—note the pooled contrast at the exit wound along the medial thigh, and a small volume at the entry wound

Limbs undergoing CT produced a series of comprehensive images as exampled in Figs. 9, 10, and 11. The limbs were imaged axially and then MPR and 3D reformats were produced from these images. The presence of contrast allowed precise delineation of the GSW track in multiple planes of view. This, alongside the measurement tools within the software package used to view the images, allowed dimensional measurement of the complete GSW tracks from each limb scanned, which are displayed as mean with standard deviation (SD) and coefficient of variation (CV) for each measurement (Table 2). Wound patterns from projectiles were observed to enter from the lateral thigh surface, traverse the posterior muscle compartment of the thigh (hamstring muscles) whilst crossing an intermuscular plane around the mid-way point, before exiting via the medial thigh surface.

**Table 2:** Mean, SD, and CV for dimensions measured on CT imaging of deer limbs post shooting

		NL			H1			D1			TT		
Projectile	CT view	Mean (mm)	SD (mm)	CV (%)	Mean (mm)	SD (mm)	CV (%)	Mean (mm)	SD (mm)	CV (%)	Mean (mm)	SD (mm)	CV (%)
7.62 mm (*n* = 4)	Axial	32.5	13.2	40.6	14.9	4.5	30.1	59.7	25.2	42.1	90.5	3.0	3.4
	Coronal	31.9	14.9	46.8	17.8	4.6	25.7	46.9	7.0	14.8	90.4	4.6	5.1

Contrast medium successfully penetrated each complete wound track to allow visualisation on CT images. CVs for NL, H1, and D1 are relatively large as would be expected due to the variability seen within GSW patterns even under controlled circumstances.

## Discussion

The different techniques examined highlight the complexities which can be found when examining GSW within an opaque model. Within this cadaveric animal limb model, the focus was on mapping the GSW track and demonstrating the behaviour of the projectile. This paper forms part of a wider programme of validation for the use of fallow deer hind limbs in ballistic research [49]. Each technique is discussed below separately.

### Flash X-ray

Flash X-ray provided information about projectile yaw but also could have been utilised to collect data on temporary cavitation, as demonstrated in previous studies [15–17, 19]. This yaw would allow for an increase in the KE delivered to the tissues and likely accounted for the larger and more variable exit wounds seen in this study. Building a dynamic picture of a GSW profile helps allow understanding of the nuances of wounds caused by different ammunition types and how one ammunition type will not always result in the same wound each time, even with conditions controlled experimentally [2]. This makes flash X-ray a versatile technique for visualising GSW patterns within opaque materials such as a cadaveric animal model. One significant disadvantage of flash X-ray use was the cost, which was relatively expensive. Flash X-ray technology also required trained expertise to operate, though was sometimes unreliable in its function. Where the time delay from foil penetration to x-ray exposure was in the order of nanoseconds, it was possible for an exposure to be mistimed, even with a very small error margin. Mistimed exposures, or a failure to trigger the x-ray, compromised samples and experiments where limbs could only sustain one GSW, resulting in additional costs to obtain more limbs to successfully test. Although the data captured was useful, the above difficulties meant that overall its sustainability within a research project would require cautious planning.

### Ultrasound

With respect to the use of ultrasound for mapping GSW tracks, the difficulties encountered outweighed the benefits. Light and portable, the use of ultrasound is versatile, and is relatively cheap; however, the variation in images compounded by gas artefact and the presence of operator dependence made it challenging to demonstrate a scientifically reproducible series of results when examining the cadaveric animal material in this study. The addition of contrast improved the quality of images gathered, as the identification of fluid within a material of fixed echogenicity is where ultrasound as an imaging technique is able to excel [21, 24, 25, 27, 28]. GSW tracks with contrast injected could be identified within the deer limbs with relative ease; however, with gas remained a confounder and there was difficulty orientating images without a reliable reference point. With tracks in excess of 100 mm, it was only possible to visualise down rather than along the track as the probe’s field of view is limited. Another crucial disadvantage for taking wounding pattern dimensional measurements was the sensitivity of the tissue to light compression by the ultrasound operator (Fig. 4), thus distorting the tissue and invalidating the accuracy of measurements. Ultrasound images, although captured with relative ease in DICOM format, also proved difficult to open on a desktop computer with compatibility issues found on multiple occasions. This made retrospective or repeat analysis challenging to manage. Owing to these difficulties and the failure to gain precise measurements, this technique was abandoned. Whilst not providing reproducible data in this study, ultrasound as a technique for imaging in ballistic research still has potential which merits further investigation.

### Dissection

Dissection was found to be of little value within this study. Although it has historically provided useful data with respect to damaged tissue excised from live animal models [37–40], its use in a cadaveric model such as this was limited due to the fact that without live tissue, determining what tissues had been damaged apart from the direct wound track was not possible. Also, measuring dimensions within the GSW pattern, apart from total track length, was challenging due to the need to directly open the wound track with a knife, which meant distorting the track. This made measurements subjective and lacking in reproducibility across the four limbs taken for dissection. Dissection had to be completed within a short timeline due to the decomposition of the cadaveric material, which in itself provided an unpleasant working environment for the researcher. This was mitigated with the researcher utilising relevant personal protective equipment (PPE) including medical gloves, goggles, and a facemask, as well as ensuring appropriate ventilation of the working area and air freshener use. Other disadvantages also included difficulty maintaining orientation throughout the respective tissue planes traversed by the projectile. The final problem was with the limb effectively being destroyed following dissection, precluding any repeat analysis, thus rendering the technique futile.

### Computed-tomography

CT of limbs following direct percutaneous injection of contrast and MPR gave demonstrable results with precise mapping of the GSW track within the samples scanned. Specific aspects of the wound patterns that were measured (as shown in Table 2) are comparable to data collected within other studies examining GSW patterns [5, 8, 10, 12, 49]. Whilst the application of CT for GSW within forensic fields is already proven [45–47], by collecting precise dimensional GSW pattern data using the method outlined in this study, contrast CT scanning offers a further tool for data capture to the ballistic researcher, particularly within otherwise opaque materials under study, e.g. animal or human tissues as opposed to gelatine. Despite these advantages, a significant disadvantage was the availability of appropriately trained personnel and limited access to the scanner itself due to pressures of clinical use. This could have potentially caused difficulty with a narrow timeline for data collection, though in this study was not an issue. Whilst no significant cost was incurred for this study due to the affiliations of authors with the institute utilised, other researchers may not be able to benefit from such an arrangement. The software for image reconstruction was also complex and required a user not only trained in its use, but also proficient with it in order to facilitate image analysis. Contrast penetration of the true wounding pattern was assumed, though it would be possible for elements of the wound profile and the distorted anatomy to prevent complete contrast penetration to all areas. This must be considered upon reviewing the images collected.

## Conclusion

Of the different techniques examined in this study, each provides merit within an appropriate scenario; however, under these test conditions, CT with contrast proved the most effective to allow precise GSW pattern analysis within a cadaveric animal limb model. These findings may be beneficial to others wishing to undertake further ballistic study both within clinical and forensic fields.

## Footnotes

1 Deer were culled for entry into the human food chain as part of planned land management, not specifically for research purposes

2 All testing was conducted at COTEC, Cranfield University
